# Commentary: Deficient Inhibition in Alcohol-Dependence: Let's Consider the Role of the Motor System!

**DOI:** 10.3389/fnins.2019.00876

**Published:** 2019-08-20

**Authors:** Lanjun Zhou, Bin Zhan, Weiqi He, Wenbo Luo

**Affiliations:** Research Center of Brain and Cognitive Neuroscience, Liaoning Normal University, Dalian, China

**Keywords:** transcranial magnetic stimulation, alcohol dependence, motor system, inhibitory control, motor evoked potentials, action preparation

Inhibitory control has a significant capacity to abort undesirable or inappropriate responses (Logan et al., 2014). As a part of executive functioning, it plays an important role in goal-directed behaviors (Luna et al., 2015). Previous literature has demonstrated that heavy drinking is related to higher impulsivity, including a reduced response inhibition (Ahmadi et al., 2013). Abstinent patients with alcohol dependence (AD) have shown impairments in response inhibition, which could increase alcohol-elicited craving (Papachristou et al., 2012), contributing to compulsive drug-seeking behaviors and enhanced relapse risk (Dalley et al., 2011).

Substantial evidence has indicated that inhibitory control depends mainly on prefrontal areas (Berlin et al., 2004; Trantham-Davidson and Chandler, 2015; Klenowski, 2018). However, the motor system may have an impact on inhibitory control. Transcranial magnetic stimulation (TMS) studies have revealed a relationship between the corticospinal excitability changes and motor inhibition in the motor system (Duque et al., 2017); therefore, motor system excitability may be suppressed during response inhibition. Research on teenagers who suffered from heavy prenatal alcohol exposure recruited less primary motor areas in an easy inhibition response task when compared with control individuals (Ware et al., 2015). This highlights the influence of motor cortex on inhibitory function.

Single pulse TMS of the primary motor cortex (M1) can measure the extent of motor inhibition, reflected by motor-evoked potentials (MEPs) (Beck et al., 2008). MEPs decrease before a motor response, which corresponds with the results showing that the motor system is inhibited during the preparation of action (Greenhouse et al., 2015a). Although impairments in inhibitory control are found in individuals with addictive behaviors, it remains unclear whether the response inhibition is modulated by motor system in alcohol use disorders during action preparation.

This hypothesis is addressed in a recent paper published in the *Neuropsychopharmacology* (Quoilin et al., 2018). In this study, the authors explored the role of motor system in inhibitory control, behavioral inhibition, and relapse in patients with AD. Their main approaches involved the collection of demographical data, including the Spielberger State Trait Anxiety Inventory, Beck Depression Inventory, and UPPS Impulsive Behavior scale, behavior tests, and a TMS procedure. Quoilin et al. hypothesized that patients with AD had reduced neural motor inhibition during action preparation. To test this hypothesis, they utilized a 115% resting motor threshold of single-pulse TMS over the non-dominant or dominant hand area of the M1 to elicit MEPs corresponding to finger muscles during an instructed-delay choice reaction time (RT) task.

The results were consistent with previous studies (Henges and Marczinski, 2012; Greenhouse et al., 2015b). Firstly, patients with AD showed a lack of behavioral inhibition and higher scores in trait impulsivity compared with the control group. Second, although suppression of MEPs was observed in the delay period relative to baseline in all participants for the forthcoming movement, the suppression for patients with AD was weaker than the controls (Greenhouse et al., 2015b; Lebon et al., 2016). Besides, both behavioral inhibition and suppression of MEPs were not associated with trait impulsivity. This supports the independence of diverse types of impulsivity, including impulsive choice (also referred to discounting of delayed rewards), impulsive action (response or motor inhibition), and impulsive personality traits (MacKillop et al., 2016). Delay discounting (but not response inhibition) is different between heavy drinking smokers and heavy drinkers only or smokers only (Moallem and Ray, 2012). Hence, the distinction of response inhibition during action preparation in different addictive groups should be addressed in the future. Finally, the authors extended these results by illustrating that the loss of inhibition was only found in relapsing patients after a year, while the persistent abstainers displayed comparable inhibitory control to healthy group in the neural and behavioral measurements. That is, a stronger disinhibition in patients indicated a higher probability of relapse. Hence, we can classify patients with AD, those prone to relapse and those who remain abstinent, and efficiently intervene through cognitive training and TMS technology to enhance their inhibitory control (Kohl et al., 2019), which may promote better rehabilitation. In addition, this is the first study to show that the paucity of inhibitory function is modulated by the motor system in patients with AD, which provides a new target of TMS for the future treatments.

Nonetheless, this study highlights some limitations. Addiction is characterized by alterations in multiple regions and brain circuits, but the effect of these related regions is not considered in the paper. In fact, the right frontotemporal (Gan et al., 2014), medial prefrontal cortex (Klenowski, 2018), and striatum (Cheng et al., 2017) are involved in inhibitory control in relevant alcohol studies; weaker functional connectivity between the frontal cortex and striatum has been found when serious alcohol users perform the response inhibition tasks (Courtney et al., 2013). Thus whether the inhibitory mechanism of M1 during action preparation depends on other brain areas should be investigated in the future. For instance, functional connectivity between M1 and other brain networks, such as the pre-supplementary motor area, or right inferior prefrontal cortex, is vital to successful inhibitory control (Duann et al., 2009). Some investigators have found that these prefrontal areas transmit information to the M1 to inhibit premature behaviors by an oscillatory beta rhythm (Picazio et al., 2014); the mechanism underlying these connections in individuals with addiction remains unknown. Combining TMS with fMRI, researchers can observe the changes of functional connectivity between M1 and other brain areas and help improve inhibitory control in patients with AD in the future. As preliminary studies, smokers can decrease their craving by real-time fMRI neurofeedback (Hartwell et al., 2016). Same technology can be applied to help the patients with AD to regulate their inhibitory control.

Besides, additional difference between persistent abstainers and relapsed patients remains unknown. For example, it is not clear whether alcohol consumption and dependence time between these two kinds of patients have significant difference. Previous research has shown that higher alcohol consumption is associated with less total brain volume (Paul et al., 2008) and neurocognitive impairment in multiple regions (Woods et al., 2016). Therefore, alcohol use history should be defined as covariate in the future. Although multiple cognitive functions are impaired in various brain regions for patients with AD, longer alcohol withdrawal period promotes functional recovery (Kopera et al., 2012). Thus, the alterations of relevant brain networks in different abstinent duration should be further investigated.

Finally, M1 mechanism of inhibitory control is also revealed by paired-pulse TMS, including short- (SICI) and long-interval intracortical inhibition (LICI), involving gamma-aminobutyric acid A (GABAA) and gamma-aminobutyric acid B (GABAB), respectively. Previous research has found that reduced LICI of dorsolateral prefrontal cortex, no difference for SICI of motor cortex in AD patients post-detoxification compared with controls (Naim-Feil et al., 2016). However, the changes on LICI and SICI of M1 in patients with AD during action preparation need to be further explored. For healthy individuals, LICI is reduced during the whole response inhibition task, while reduced SICI is only found in informative cues (Cirillo et al., 2018); Individuals with better improvement in motor training show a reduction in GABAergic release in movement preparation (Dupont-Hadwen et al., 2019). Hence, Combining TMS and motor training, researchers should further investigate how to improve inhibitory control in patients with AD.

In conclusion, the current work explored the different capacities for inhibition between patients with AD and healthy controls, including neural motor, behavior, and trait impulsivity. These researchers discovered that patients with AD had reduced motor cortex excitability and higher trait impulsivity compared with the controls. In addition, they reported a dysfunction in the neural inhibitory ability of patients with AD during movement preparation, especially in patients who had relapsed one year later. These findings have revealed the importance of inhibitory processes in forthcoming actions to healthy individuals. These data suggest that improved inhibitory control plays a significant role in preventing a relapse in serious alcoholism. Using TMS over the related motor cortex to modify inhibitory processes may be a prospective treatment for patients with addiction (Dupont-Hadwen et al., 2019).

## Author Contributions

LZ, WL, and WH conceived the idea, revised all the literature, and wrote the manuscript. BZ read and revised the manuscript. WH and WL contributed to the revision of the manuscript. All authors read and approved the submitted version.

### Conflict of Interest Statement

The authors declare that the research was conducted in the absence of any commercial or financial relationships that could be construed as a potential conflict of interest.
